# Special Notice

**Published:** 1900-04

**Authors:** 


### SPECIAL NOTICE.

Word has been received at this office that some one in Illinois has been representing himself as an agent to collect subscriptions for the International Dental Journal. Subscribers are notified that no one is authorized to take subscriptions in that State.

